# Subjective Cognitive Complaints and Symptom Severity in Patients With Borderline Personality Disorder

**DOI:** 10.62641/aep.v53i5.1933

**Published:** 2025-10-05

**Authors:** Elsa Carolina Muñoz-Toledo, Alejandra Mondragón-Maya, Iván Arango, Ana Fresán, María Yoldi-Negrete, Juan José Sánchez-Sosa

**Affiliations:** ^1^Faculty of Psychology, Ciudad Universitaria, National Autonomous University of Mexico, 04510 Mexico City, Mexico; ^2^Faculty of Superior Studies Iztacala, National Autonomous University of Mexico, 54090 Tlalnepantla de Baz, State of Mexico, Mexico; ^3^Borderline Personality Disorder Clinic, Ramon de la Fuente Muñiz National Institute of Psychiatry, 14370 Mexico City, Mexico; ^4^Clinical Epidemiology Laboratory, Subdirectorate of Clinical Research, Ramon de la Fuente Muñiz National Institute of Psychiatry, 14370 Mexico City, Mexico; ^5^Faculty of Psychology, Ciudad Universitaria, National Autonomous University of Mexico, 04510 Mexico City, Mexico

**Keywords:** subjective cognitive complaints, symptom severity, borderline personality disorder, BPD

## Abstract

**Background:**

Borderline Personality Disorder (BPD) may include rigid thoughts, interpersonal difficulties, unstable identity, and the presentation of intense and volatile emotions; the leading indicator for this condition is symptom severity (SS). Within this spectrum, there could be subjective cognitive complaints (SCC) that may worsen the severity of the clinical condition. The present study aimed to compare SCC scores between BPD participants and a group of control subjects (CS) and to determine the association between BPD symptom severity and SCC in participants with BPD.

**Methods:**

102 participants with BPD and 73 CS participants from Ramon de la Fuente Muñiz National Institute of Psychiaty were included. In order to measure symptom severity, participants responded to the Borderline Evaluation of Severity Over Time (BEST) scale, and for SCC through the Cognitive Complaints in Bipolar Disorder Rating Assessment (COBRA) scale.

**Results:**

COBRA total scores were higher in participants with BPD (26.9 ± 9.3) compared to CS (12.9 ± 6.8; t = –10.8, *p* < 0.001). Positive correlations resulted between the COBRA total score and the BEST dimensions of thoughts and feelings (r = 0.38, *p* < 0.001), Negative behaviors (r = 0.30, *p* < 0.002), and the Total BEST score (r = 0.38, *p* < 0.001).

**Conclusion:**

Participants of this study experience high levels of SS, which includes the constant presence of harmful and intrusive thoughts and feelings, as well as dysfunctional behaviors. This clinical context correlates positively with a high report of SCC, thus highlighting the importance of evaluating SCC as a relevant factor of patients’ clinical experience and considering it within their personal needs to improve the course and prognosis throughout their treatment.

## Introduction

Borderline personality disorder (BPD) is a clinical condition characterized by an inflexible and persistent pattern of interpersonal difficulties, along with dichotomic thoughts that fluctuate between idealization and devaluation of oneself and others, identity disorders, as well as the presence of sudden intense emotions that could generate impulsive, risky and/or self-harm behaviors [1, 2]. In some cases, paranoid ideation, dissociative symptoms, and/or suicidal behavior can be present [3, 4].

Due to symptomatic chronicity, BPD is considered a psychiatric condition that causes significant distress in the individuals who suffer from it, thus generating a negative impact in distinct aspects of life, for example, through low levels of self-perceived satisfaction, difficulties in adapting to goal-directed lifestyles [5, 6, 7], problems in the occupational field [8, 9], as well as a series of neurocognitive dysfunctions that impact BPD patients’ performance and daily functioning [10, 11, 12]. Some studies report disturbances in attention, memory, visuospatial processing, and executive functions. Such deficits generally include high subjective cognitive complaints (SCC) [12, 13, 14].

Although barely explored in the psychiatric setting, SCC involves excessive concern, usually experienced by middle-aged adults, related to one’s own memory and attention functioning (among other cognitive domains) and later progressive deterioration [15, 16]. Recent results show an inconsistent association between SCC and objective scores from specialized cognitive instruments [17, 18].

BPD patients tend to show high levels of SCC, usually observed as concerns related to memory and cognitive performance and the inability to accomplish daily living activities [18, 19]. These complaints do not objectively correspond with the neuropsychological assessment performance, although a dysfunctional neuropsychological profile can be present [20, 21]; that is, the frequency and intensity of the SCC reported by these patients tend to be higher than the outcomes from objective neuropsychological measures, even if neurocognitive deficits are present.

As described above, exploring these variables has focused on the relationship between specialized professionals’ neuropsychological assessments and self-reporting SCC measures, in which no lineal association seems to exist. However, exploring the relationship between SCC and clinical variables related to the psychiatric condition, like symptomatic severity, is also relevant. In this regard, the findings are inconsistent. While some authors have found a relationship between SCC levels and the presence of mood and emotional dysregulation symptomatology [18, 22, 23], others report no association between such variables, suggesting that SCC intensity could be more related to sociodemographic variables like sex, age, or educational achievement [16, 24, 25].

As mentioned before, BPD symptomatology severity plays a significant negative role in the quality of life and functionality of the patients who suffer from it [7, 8, 9], and the perception of cognitive disturbances cannot be disregarded in its impact on daily living of patients with BPD [18, 19]. Some authors have reported an association between SCC and the presence of clinical symptomatology in these patients [26, 27, 28, 29], whilst others have suggested that the highly intrusive nature of SCC could decrease the patient’s motivation to change and engage with their pharmacological and psychotherapeutic treatment [16, 28], thus complicating the course of the disorder and preventing the patients’ adequate psychosocial reintegration and quality of life improvement.

Thus, the combined presence of symptomatic severity and SCC in BPD patients could generate a feedback process that stimulates the presence and maintenance of BPD clinical features [20, 28, 29]. Thus, exploring the possible association between BPD symptomatic severity and SCC has become relevant for research, especially for course and prognosis purposes. Therefore, the aims of the present study were (1) to compare SCC scores between BPD subjects and a group of control subjects (CS) and (2) to determine the association between BPD symptom severity and SCC in participants with BPD.

## Methods

### Participants

The study employed a non-experimental, cross-sectional, correlational design to examine the relationships between SCC and borderline personality symptom severity at a single point in time. The study’s cross-sectional nature means that data was collected from participants (subjects with BPD and CS) at one specific moment. The present design allows the analysis of associations without manipulating any of the variables, allowing the identification of potential links between the studied variables without inferring causality.

### Subjects With BPD

Outpatients with a confirmed diagnosis of BPD according to International Classification of Diseases 11th Revision (ICD-11) criteria [30] were recruited at the BPD Clinic from the Ramon de la Fuente Muñiz National Institute of Psychiatry in Mexico City from January 2023 to February 2024. Participants received BPD diagnosis from specialized psychiatrists through a clinical interview based on ICD-11 [30] diagnostic criteria and The Structured Clinical Interview for Diagnostic and Statistical Manual of Mental Disorders, Fourth Edition Axis II Personality Disorders [31, 32]. Patients participated in the study if they complied with the following inclusion criteria: (1) men or women, (2) minimum age of 18 years at the time of the study, (3) currently being treated at the BPD Clinic, and (4) voluntarily accepted to participate. Exclusion criteria were as follows: (a) patients with severe Personality Disorder with a predominance of dissocial traits, (b) the presence of active psychotic symptoms, bipolar disorder, neurodevelopmental disorders, and/or substance abuse disorder without remission for at least three months, and (c) patients who had suffered from COVID-19 and reported cognitive difficulties following the infection.

### Control Subjects (CS)

Individuals from this group were taken from the dataset of the Cognitive Complaints in Bipolar Disorder Rating Assessment (COBRA) scale validation study [33]. Recruiting this sample involved a convenience sample approach with subjects from the general population who were available and willing to participate. Subjects were screened with a face-to-face interview with a psychiatrist for Axis-I disorders. Those who self-reported any Axis-1 disorder, or received attention in a psychiatric facility did not participate in the study. We aimed to include the highest possible number of control subjects matched by sex and age with the BPD group.

### Measures and Instruments

Demographic features assessed in both groups through a face-to-face interview included Sex (man/woman), current age, years of schooling, and occupation (employed/housewife-student/none).

The COBRA is an instrument that measures SCC on attention, memory, and executive functioning domains in patients with bipolar disorder. It is a 16-item self-report instrument with a 4-point Likert-type scale ranging from “0 = Never” to “3 = All the time”. The total score results from the sum of each item score, ranging between 0 and 48 points. Higher scores indicate more subjective complaints. The study applied the adapted version for the Mexican population since it has shown adequate psychometric properties (i.e., total internal consistency of 0.91 and adequate discriminant validity) [33]. We assessed internal consistency for the group of participants with BPD; Cronbach’s alpha value was 0.89 for this group and the CS 0.87, indicating its adequacy for use in this study.

The Borderline Evaluation of Severity Over Time (BEST) scale determines symptomatic severity. It is a 15-item instrument with a 4-point Likert-type scale from “None/slight” to “Extreme” or “Almost never” to “Almost always”. It is a self-report scale that comprises 3 subscales which assess: (A) Thoughts and feelings, with 8 items and a response range within 8–40 points measuring extreme and dichotomous cognitions, as well as emotions such as anger and feelings of emptiness; (B) Negative behaviors, with 4 items and response range between 4–20 assessing dysfunctional actions, such as self-harm or impulsive behavior; and (C) Positive/self-care behaviors, composed by 3 items with scores between 3–15 that evaluates an adequate level of treatment adherence as well as leisure behaviors. The total score resulted from adding the scores from subscales A and B. The total score from subscale C gets subtracted and includes a correction factor of 15. As a result, the total score range is from 15 to 74 points. The Spanish version was employed since it achieved a total Cronbach’s alpha of 0.80, thus demonstrating adequate internal consistency [34].

### Procedure

Participants responded to the instruments as part of the regular admissions protocol to the BPD Clinic at Ramon de la Fuente Muñiz National Institute of Psychiaty. Medical residents in their third and fourth years of psychiatric specialty training contacted and scheduled appointments with outpatients who were registered on a waiting list to be admitted to the BPD Clinic.

Such appointments were in person or individual and aimed to corroborate or dismiss a BPD diagnosis. Once the BPD diagnosis was confirmed with the clinical interview and diagnostic guidelines and instruments [30, 31, 32], participants completed the BEST and COBRA assessments. After completing the instruments, all participants were thanked for their participation and continued their treatment at the BPD Clinic.

### Statistical Analysis

Descriptive analyses involved determining frequencies and percentages for categorical variables and mean and standard deviations for continuous variables. Chi-square tests (χ^2^) and independent sample *t*-tests allowed the comparison of demographic features, COBRA items, and total scores between BPD participants and control subjects. The Kolmogorov-Smirnov test indicated a normal data distribution for the COBRA scale (*p* = 0.09) and the BEST scale (*p* = 0.20). Additionally, according to Levene’s test, the variances of both instruments were homogeneous (>0.05), and the scores from both scales showed a significant correlation (r = 0.38). The demographic variables were considered covariables, and the comparison mean change in the COBRA total score between groups considered the covariables effect or interaction (group × covariable). Considering these assumptions, demographic variables where significant differences arose between groups were included in a general linear analysis using Analysis of Covariance (ANCOVA) modeling. These demographic variables were included as covariables, and the mean change in the COBRA total score between groups was compared, considering the covariable effect or interaction (group × covariable) to determine their influence on the comparison of the COBRA total score between groups. Finally, the Pearson correlation coefficient determined the linear association between the COBRA total score and the symptom severity dimensions and total score of the BEST scale. All analyses used SPSS Statistics (IBM Inc., Armonk, NY, USA) version 21 for Windows, PC. the alpha value for tests was set at *p* ≤ 0.05.

### Ethical Considerations

The pertinent Ethics and Research Committees approved the procedures and methods of the present study (CEI/C/032/2022 register number) from the Ramon de la Fuente Muñiz National Institute of Psychiaty, following the ethical principles and guidelines of the Declaration of Helsinki. All participants volunteered to be included in the study after listening to detailed explanations of the study’s nature and procedures, guaranteeing data confidentiality, and ensuring the exclusive use of the obtained information for research purposes. Once the participants verbally agreed to be included in the study, they signed the respective informed consent before initiating the assessments. None of the participants received any economic compensation for participating in the study.

## Results

### Demographic Features

The analysis included a total of 73 CS and 102 subjects with BPD. Women accounted for more than 80% of both groups (CS 80.8%, n = 59 vs. BPD 90.2%, n = 92; *p* = 0.07). Current occupation was similar among groups, 58.9% (n = 43) and 53.9% (n = 55) had a paid work, followed by those with unpaid work (CS, 31.5%, n = 23; BPD, 28.4%, n = 29) and lastly, 9.6% (n = 7) of the CS and 17.6% (n = 18) of the BPD participants were unemployed (*p* = 0.32). Participants with BPD were younger (27.5 ± 8.4 years vs. 30.2 ± 6.1 years; *p* = 0.02) and with less years of schooling than CS (15.0 ± 5.0 years vs. 16.4 ± 2.4; *p* = 0.02).

### Subjective Cognitive Complaints

The COBRA total score differed between CS and BPD participants. The score of BPD subjects (26.9 ± 9.3) was much higher than that reported by the CS group (12.9 ± 6.8; t = –10.8, *p* < 0.001), reflecting much more SCC in the BPD group. Age and education level acted as covariates based on the differences observed in these variables between groups. According to the general linear analysis with the ANCOVA modeling, the difference between groups on the COBRA total score was neither influenced by age (group × age: F = 1.5, *p* = 0.12) nor by years of schooling (group × schooling: F = 1.3, *p* = 0.13). Considering the individual COBRA items, BPD participants report specific cognitive complaints more frequently than the participants of the CS group (see Table 1).

**Table 1.** **Comparison of the frequency of presentation of specific cognitive complaints among groups**.

COBRA items ^a⁢b^	Control subjects		BPD subjects	
	n = 73		n = 102	
	n	%	n	%
1. Do you have difficulties to remember peoples’ names?	13	17.8	39	38.2
2. Do you have difficulties to find objects of daily use (keys, glasses, wristwatch…)?	5	6.8	61	59.8
3. Do you find it difficult to remember situations that were important to you?	4	5.5	50	49.0
4. Is it hard for you to place important events in time?	13	17.8	58	56.9
5. Do you find it hard to concentrate when reading a book or a newspaper?	15	20.5	68	66.7
6. Do you have problems recalling what you have read or have been told recently?	11	15.1	62	60.8
7. Do you have the feeling that you do not finish what you begin?	19	26.0	74	72.5
8. Does it take you longer than normal to complete your daily tasks?	5	6.8	53	52.0
9. Have you ever felt disoriented in the street?	8	11.0	35	34.3
10. When people remind you of a conversation or a comment you heard, do you get the impression that it is the first time you hear it?	7	9.6	43	42.2
11. Is it sometimes difficult for you to find the words to express your ideas?	13	17.8	62	60.8
12. Are you easily distracted?	30	41.1	85	83.3
13. Do you find it hard to do simple mental calculations?	14	19.2	52	51.0
14. Do you get the impression that you cannot follow a conversation?	9	12.3	58	56.9
15. Have you noticed that you find it difficult to learn new information?	7	9.6	50	49.0
16. Do you struggle to keep focused on a particular task for a long time?	21	28.8	71	70.3

^a^ The table only presents the frequency and percentage of item response “frequently/always”. ^b^ All item comparisons deemed significant at a *p* < 0.01.

The BEST dimensions and total score were as follows: thoughts and feelings (28.0 ± 7.3), negative behaviors (11.0 ± 0.2), positive behaviors (10.0 ± 2.7), and total score (29.0 ± 11.7). These scores reflect moderate to severe symptomatology in the participants assessed.

**Fig. 1.**
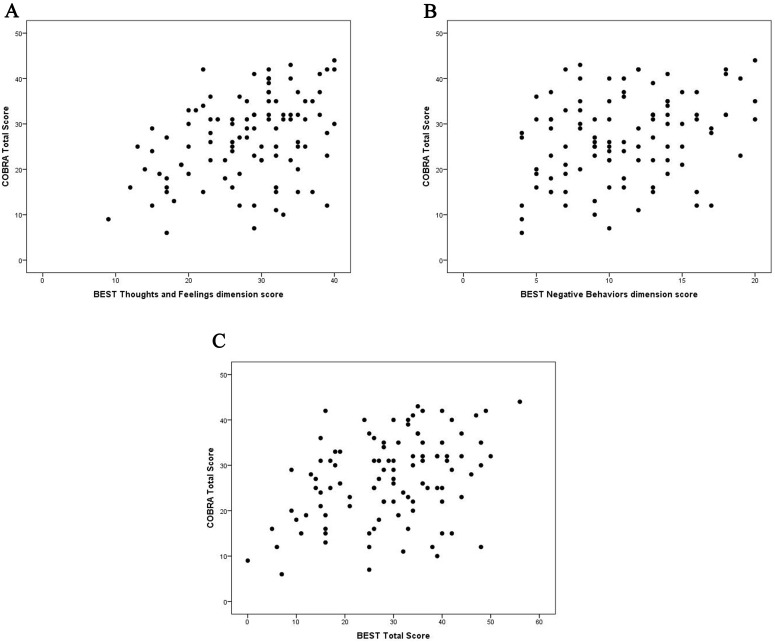
**COBRA score correlation with the BEST dimensions and total score**. (A) COBRA score correlation with the BEST thoughts and feelings dimension score. (B) COBRA score correlation with the BEST negative behaviors dimension score. (C) COBRA score correlation with the BEST total score.

A positive correlation resulted between the COBRA score and the BEST dimensions of thoughts and feelings (r = 0.38, *p* < 0.001), negative behaviors (r = 0.30, *p* < 0.002), and the total BEST score (r = 0.38, *p* < 0.001). These results imply that higher symptom severity correlates with higher SCC. There was no correlation between the COBRA score and the BEST Positive behaviors dimension (see Fig. 1A–C).

## Discussion 

SCC reports from BPD patients are high and associated with concerns regarding memory, cognitive performance, and an inability to successfully perform daily activities. Within this context, the present study aimed to compare the COBRA scores between BPD patients and a group of CS and to determine the association between BPD symptom severity and SCC in participants with BPD.

Worldwide, BPD is the most frequent diagnosis within the personality disorder spectrum, and it is more prevalent in middle-aged women [1, 2]. Mexico is not the exception, as shown by the present study, in which 90.2% of the participants with BPD were women. Another sociodemographic aspect that deserves underscoring is the high educational level of our sample of BPD patients. They only differed with the CS by one year. Our BPD patients who attended the Ramon de la Fuente Muñiz National Institute of Psychiaty (a public health service facility of highly specialized third-level healthcare located in the country’s biggest and most important city) are highly educated. Data from this study contrasts with international findings, which usually find that BPD patients tend to have low levels of educational attainment.

Regarding the comparison analysis, significant differences between groups were observed in the COBRA scores, whereas BPD patients scored higher. Whilst the scores from CS tended to be lower, indicating that the reported answers from this group mainly corresponded to the “Never” or “Sometimes” categories, BPD patients reported higher and more significant difficulties and cognitive deficits, by choosing mostly the “Always” category. These results involve relevant clinical implications since they could reflect a “distorted” self-perception of the patients’ cognitive symptoms [12, 13, 14].

Although specialized professionals have demonstrated deficits in the neuropsychological profile of these patients, such dysfunctions do not necessarily match the high SCC levels that patients report [20, 21]. Such “distorted” perception could be the result of the clinical core features of BPD instead of a mere exaggeration of the subjective report. One of the leading indicators of the borderline pattern [1, 2, 3, 4], as resumed by the ICD-11 [30], refers to a cognitive instability pattern that tends to be dichotomic and extremist. Patients with BPD tend to polarize their points of view, opinions, and perceptions regarding themselves, others, and the environment, so the total scores obtained from COBRA in the present study could reflect such thought patterns. Moreover, in clinical and individual terms, such results could also reflect that patients genuinely suffer from the cognitive symptoms they perceive. Also, it is probable that their perception of having such cognitive deficiencies further increases a negative perspective of themselves.

The second aim of the present study was to analyze the correlations between SCC and symptomatic severity in the BPD group. Results showed significant positive correlations between COBRA total score, BEST’s subscales of thoughts and feelings, and Negative behaviors, as well as the total score, thus indicating that the higher the severity of the symptoms, the higher SCC experienced. From a clinical viewpoint, it is evident that symptomatic severity in BPD patients significantly permeates and impacts their functional, cognitive, and affective abilities, as well as their capacity to interact with others and adapt to their surrounding socio-cultural context.

Remarkably, the results of this study showed correlations between two of the BEST subscales that represent dysfunctional symptoms. The first of them, i.e., thoughts and feelings, refer to BPD patient’s internal experiences when interacting with their environment; that is, the subscale evaluates dichotomous, extreme, and absolutist cognitions and opinions, as well as emotions such as anger, feelings of emptiness and suicidal ideation. In general, the higher the score, the more distress patients perceive. The second of them assesses negative behaviors. This subscale allows quantifying dysfunctional and ineffective behaviors, such as self-harm, impulsive behavior, or extremist acts. One of the possible explanations for why these two subscales are associated with SCC could be the discomfort and suffering experienced by patients. If symptom severity reported by BPD patients reflects extremist, dichotomous thoughts and dysfunctional behaviors, this could also be impacting the auto-perception of their performances in cognitive domains.

By contrast, no correlations resulted between SCC and positive behaviors; the third BEST subscale measures behavioral indicators of the use of skills learned in treatment. A possible explanation could be that the lack of association with this scale could be more related to the degree of the patients’ commitment to adhering to and improving their care behaviors, regardless of the subjective perception of their performance. This could reflect patients’ difficulties overcoming discomfort during their daily activities. For example, some studies suggest that BPD patients perceive poor personal performance in carrying out daily activities in their work environment and household activities. Also, they report difficulties moving from one point to another within their home residence [18, 19, 20, 21].

The results from the present study support the importance of considering the symptomatic severity level and its relationship with other clinical variables as a priority when making decisions related to effective interventions focused on the patient’s needs, serving as a starting point regarding the implications for the patient in their daily living, as well as in their treatment and improvement prognosis. Precisely, the relationship that we found between symptomatic severity and SCC may imply a clinical key point since the increase of any of these variables (e.g., increased SCC) could be an indicator that other clinical variables may be increased as well (e.g., higher symptom severity), thus affecting the perception of other severe symptoms related to the disorder, like impulsive or risky behavior [1, 2, 3, 4].

Moreover, the results of the present study also raise technical implications. SCC assessment is relatively simple, and obtaining information on this nature at the initial phases of the disorder could help mental health professionals identify and manage appropriately the frequent symptomatology and breakdown episodes in BPD. The results of the present study highlight the importance of the continuous assessment of SCC in BPD.

Some limitations when interpreting the results of our study are apparent. First, data were collected through self-report instruments. Both instruments have shown adequate psychometric properties [33, 34]. Furthermore, they are practical, simple to use, and provide relevant clinical information. However, the nature of self-report instruments could lead to biased reports. In this regard, we recommend using instruments applicable by specialized professionals (in addition to clinical interviews) to corroborate the obtained information. Second, the present study used a cross-sectional design, so causal relationships among variables could not be evaluated. It is important to include longitudinal designs with follow-up phases that could measure the fluctuation over time of the symptomatology and SCC, thus assessing if the relationship between both variables remains significant throughout the disorder. Third, the Ramon de la Fuente Muñiz National Institute of Psychiaty patients included in this study had a high educational level, which differs from several reports in the international research literature. This sociodemographic characteristic could be a limitation when comparing results with other BPD patients worldwide. Finally, our BPD patients presented moderate to severe symptomatology, so it would be important to assess different range symptomatology levels to explore how patients experience SCC when symptom severity is lower.

## Conclusion

The results of the present study highlight the importance of SCC assessment as a core feature within the BPD population. Previously, the lack of correspondence between objective neuropsychological assessments conducted by specialized professionals and self-reported SCC promoted the devaluation of the latter. However, the results of the present study show that SCC in BPD patients tends to be intense, distressful, and concerning, unlike what we observed in CS. Moreover, SCC is associated with symptom severity so that it may be a potential indicator of clinical outcome in BPD patients. Thus, this line of research certainly requires further clinical studies.

## Availability of Data and Materials

The datasets generated and/or analyzed during the current study are not publicly available due to privacy and ethical restrictions, but are available from the corresponding author on reasonable request, subject to approval by respective Ethics Commitee.
